# An investigation into the prevalence of sleep disturbances in primary Sjögren’s syndrome: a systematic review of the literature

**DOI:** 10.1093/rheumatology/kew443

**Published:** 2016-12-24

**Authors:** Katie L. Hackett, Zoe M. Gotts, Jason Ellis, Vincent Deary, Tim Rapley, Wan-Fai Ng, Julia L. Newton, Katherine H. O. Deane

**Affiliations:** 1 Musculoskeletal Research Group, Institute of Cellular Medicine & NIHR Biomedical Research Centre for Ageing and Chronic Diseases; 2 Newcastle upon Tyne Hospitals NHS Foundation Trust; 3 Institute of Health and Society, Newcastle University; 4 Northumbria Centre for Sleep Research, Faculty of Health and Life Sciences, Northumbria University; 5 Faculty of Health and Life Sciences, Northumbria University; 6 Institute of Cellular Medicine & NIHR Biomedical Research Centre for Ageing and Chronic Diseases, Newcastle upon Tyne; 7 School of Health Sciences, University of East Anglia, Norwich, UK

**Keywords:** Sjögren’s syndrome, systematic review, sleep, quality of life, disability evaluation

## Abstract

**Objectives.** To identify whether sleep disturbances are more prevalent in primary SS (pSS) patients compared with the general population and to recognize which specific sleep symptoms are particularly problematic in this population.

**Methods.** Electronic searches of the literature were conducted in PubMed, Medline (Ovid), Embase (Ovid), PsychINFO (Ovid) and Web of Science and the search strategy registered *a priori*. Titles and abstracts were reviewed by two authors independently against a set of prespecified inclusion/exclusion criteria, reference lists were examined and a narrative synthesis of the included articles was conducted.

**Results.** Eight whole-text papers containing nine separate studies met the inclusion criteria and were included in the narrative analysis. Few of these studies met all of the quality assessment criteria. The studies used a range of self-reported measures and objective measures, including polysomnography. Mixed evidence was obtained for some of the individual sleep outcomes, but overall compared with controls, pSS patients reported greater subjective sleep disturbances and daytime somnolence and demonstrated more night awakenings and pre-existing obstructive sleep apnoea.

**Conclusions.** A range of sleep disturbances are commonly reported in pSS patients. Further polysomnography studies are recommended to confirm the increased prevalence of night awakenings and obstructive sleep apnoea in this patient group. pSS patients with excessive daytime somnolence should be screened for co-morbid sleep disorders and treated appropriately. Interventions targeted at sleep difficulties in pSS, such as cognitive behavioural therapy for insomnia and nocturnal humidification devices, have the potential to improve quality of life in this patient group and warrant further investigation.

Rheumatology key messages

- Sleep disturbances are common in primary SS patients and should be identified and treated appropriately.
- Interventions targeted at sleep difficulties in primary SS warrant further investigation.

## Introduction

Primary SS (pSS) is a systemic autoimmune disease characterized by sicca symptoms [1]. Extraglandular features are commonly seen in pSS patients, including fatigue [2], orthostatic intolerance [3], pain [4] and depression [5]. These patients commonly experience impaired function [6] and poor health-related quality of life [8]. Fatigue is seen in 75% of patients with pSS [7], is strongly correlated with poor quality of life [8–10] and is associated with functional impairment [6]. Due to the prevalence and impact of fatigue, there has been much research into factors associated with this symptom, including potential genetic associations [11] and anti-inflammatory mechanisms [12]. Sleep disturbances have also been reported in the pSS literature [13] and are associated with fatigue [2]. In the general population, impaired sleep is associated with adverse health outcomes including weight gain, depression, pain, impaired immune function, impaired functional performance, increased risk of early mortality and cognitive symptoms such as increased errors and increased risk of accidents [14]. Current recommendations are that adults should regularly have between 7 and 9 h of sleep consistently per night [14].

Many sleep disturbances are potentially modifiable [15–18]. Therefore the successful identification and treatment of sleep problems may have a positive effect on symptoms such as pain, mood and fatigue, resulting in improvements in physical and cognitive functioning and quality of life.

A previous review of sleep disturbances in rheumatological diseases included pSS [19], but this review was published some time ago and the pSS section was predominantly based on one comparative study that used Rheumatoid arthritis (RA) patients as controls. Thus an up-to-date systematic review of the pSS sleep literature, including normative data on healthy controls, is required.

The aim of this review was to identify all the published literature on sleep difficulties in pSS in order to answer the following questions: Are sleep difficulties more prevalent in pSS patients than in the general population and which sleep difficulties are more prevalent in pSS patients than in the general population?

## Methods

A systematic review of the published literature on sleep and pSS was conducted. The protocol was published prospectively with PROSPERO, an international prospective register of systematic reviews (CRD42015024977) [20]. The methodological framework used was the Preferred Reporting Items for Systematic Reviews and Meta-analyses statement [21].

## Eligibility criteria

Eligible studies were English-language primary research papers published in full. These included intervention, diagnostic, prognostic and aetiological studies with adult participants (>18 years) with a diagnosis of pSS. Case studies and review papers were excluded. Where papers report mixed populations, only studies that analysed the pSS population separately were included. Outcomes had to include sleep outcomes and other outcomes that have a relationship with sleep. In mixed population studies, pSS data had to be reported separately for pSS patients. Data for pSS had to be compared with a control population, which could be healthy controls or controls with other diseases. Therefore studies that did not compare data from a pSS group with a non-pSS control group were excluded from this review.

## Search strategy

Databases [PubMed, Medline (Ovid), Embase (Ovid), PsychINFO (Ovid) and Web of Science] were searched from inception to September 2015 using a prespecified search string (supplementary Table 1, available at *Rheumatology* Online). The references of all included studies were also searched. Two reviewers (K.H. and Z.G.) independently examined the titles and abstracts of all records identified and full papers were retrieved for all papers that met inclusion criteria. All full-text articles were screened by two review authors independently (K.H. and K.D.) for inclusion.

## Data extraction and quality assessment

Data were extracted by one author (K.H.) onto a piloted form. These were checked by a second author (K.D.). Risk of bias was assessed at the study level separately by two authors (K.H. and K.D.) using the Joanna Briggs Institute Prevalence Critical Appraisal Tool [22] and specific notes for questions within the tool were agreed upon between the authors (K.H. and K.D.) to reduce ambiguity prior to making a decision for each criteria (supplementary Table 2, available at *Rheumatology* Online). Disagreements between reviewers were resolved through discussion.

## Summary measures

Any sleep summary measure that compared a pSS cohort with a comparative group was extracted. These include differences in means and medians and odds ratios. Data were combined in a narrative synthesis due to the expected heterogeneity of the included studies.

## Results

**Fig. 1:**
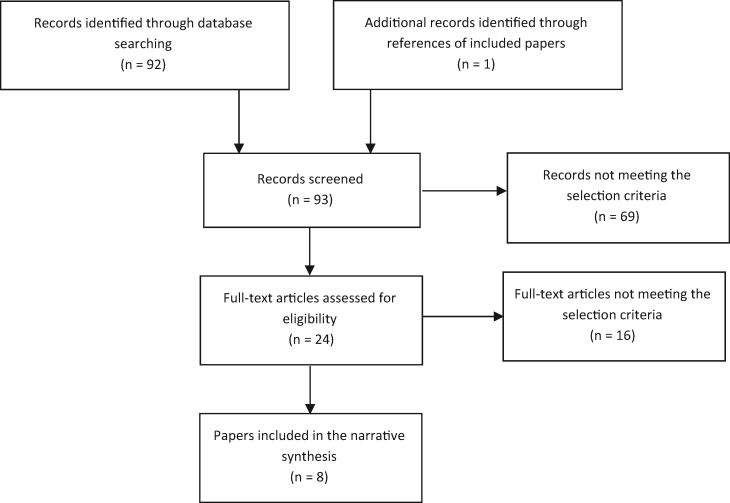
Flow diagram of articles included in this review

Nine studies from eight publications were identified for inclusion in this narrative review [13, 23–29] (Fig. 1). A summary of the included studies are in Table 1. Sixteen studies did not meet the inclusion criteria. Excluded studies with reasons for exclusion can be found in supplementary Table 3, available at *Rheumatology* Online. One excluded study was a small uncontrolled study (*n* = 9) of a nocturnal humidification device that reduced nocturnal sicca symptoms in the participants [30]. Another excluded study included the use of an artificial saliva water spray, compared with placebo, to improve nocturnal oral dryness symptoms, as improvements were demonstrated in both the placebo and intervention groups [31]. This review did not set out to investigate interventions for pSS sleep disturbances, but these findings are considered in the context of potential future interventions in the discussion.

**Table 1:** Summary of included studies

Author, year and country	Study design	Participants	Sleep outcomes
Goodchild *et al.*, 2010, UK [13]	Observational prospective study	pSS: *n* = 14, AECG diagnostic criteria for pSS [29], 100% female, mean age 58 years, MDD 13 years	Sleep diary
		RA: Rheumatoid arthritis *n* = 25, 100% female, mean age 62 years, MDD 9 years	Actigraphy
Gudbjörnsson *et al.*, 1993, Sweden [23]	Study 1: cross-sectional sleep questionnaireStudy 2: observational study Polysomnography for two consecutive nights	Study 1:	Study 1: Uppsala Sleep Inventory [34]
		pSS: *n* = 40, Copenhagen diagnostic criteria for pSS [32], 95% female, mean age 53 years. Ten pSS patients also had PSG	Study 2: polysomnography
		RA: *n* = 42, ARA criteria for classical RA: Rheumatoid arthritis [33], 100% female, 10 had symptoms of secondary SS	
		HC: *n* = 60, 100% female, age matched with the pSS participants	
		Study 2:	
		pSS: *n* = 10, no demographic information provided	
		HC: *n* = 30, middle-aged	
Hilditch *et al.*, 2008, Australia [24]	Observational study over a night’s sleep	pSS: *n* = 11, AECG diagnostic criteria for pSS, 100% female, mean age 61 years, MDD not reported	Electroencephalogram, electrooculogram, submental electromyogram; respiratory (inspiratory flow, end-tidal CO_2_ and mask leak); breathing effort; upper airway collapsibility; oral wetness and saliva surface tension
		HC: *n* = 8, all female, mean age 55.9 years, age matched with patient group	
Theander *et al.*, 2010, Sweden [25]	Cross-sectional survey	pSS: *n* = 77, AECG criteria for pSS, 90% female, median age 61 years, MDD 12 years	Epworth Sleepiness Scale [35], Restless Legs Syndrome Questionnaire [36], Lund University Sleep Questionnaire [25], Profile of Fatigue, fatigue VAS
		HC: *n* = 59, 90% female, median age 55 years	
Tishler *et al.*, 1997, Israel [26]	Cross-sectional survey	pSS: *n* = 65, AECG classification criteria for pSS, 92% female, mean age 57.3 years, MDD 8.3 years	Mini Sleep Questionnaire [26]
		RA (group A): *n* = 67, 83% female, MDD 12.6 years	
		RA with sicca symptoms (group B): *n* = 63, 70% female, MDD 15.1 years	
		OA: *n* = 31, 94% female, MDD 10.3 years	
Usmani *et al.*, 2012, Australia [27]	Observational study	pSS: *n* = 28, 100% female, AECG classification criteria for pSS, mean age 58.7 years, MDD not stated	Epworth Sleepiness Scale, polysomnography, Apnoea-Hypopnea Index [27]
		HC: *n* = 18, 100% female, mean age 55.8 years	
van Oers *et al.*, 2010, Netherlands [28]	Repeated measures study to compare variability of fatigue during the day	pSS: *n* = 29, 100% female, AECG criteria for pSS, mean age 53.3 years, MDD not stated	15-item Dutch questionnaire on sleep quality [37]
		SLE: *n* = 23, 100% female	
		RA: *n* = 19, 100% female	
		HC: *n* = 52, 100% female, mean age 51 years	
Walker *et al.*, 2003, Australia [29]	Compared differences in urinary symptoms and daytime sleepiness	pSS: *n* = 76, European Community criteria for pSS, 100% female, median age 58 years, MDD not stated	Epworth Sleepiness Scale, FACIT-F [38], American Urological Symptom Index [39]
		OA: *n* = 43, 100% female, median age 64 years	

AECG: American-European Consensus Group Criteria; ARA: American Rheumatism Association; HC: healthy controls; MDD: mean disease duration; OSA: obstructive sleep apnoea; VAS: visual analogue scale.

A total of 93 records were screened. Most studies were excluded at the title stage, as they were not relevant to the review or did not fit the inclusion criteria (Fig. 1). Fourteen publications were examined in more detail before exclusion (supplementary Table S1, available at *Rheumatology* Online) and nine studies from eight publications were included in this narrative review [13, 23–29] (Table 1). Gudbjörnsson *et al.* [23] included two studies in their paper and these are referred to as Gudbjörnsson *et al.* study 1 (a comparative study of sleep symptoms in three populations) and study 2 (a polysomnography study) in this review for clarity.

## Assessment of bias

The risk of bias quality assessment findings of the included studies are presented in Table 2. Three studies included only female pSS participants [13, 24, 29] and were consequently deemed as not being representative of the target population. Two studies (Gudbjörnsson *et al.* studies 1 and 2) [23] used the Copenhagen Classification Criteria [32] to identify their subjects. These criteria are not validated or accepted universally [40], therefore these studies were also scored as not being representative of the target population. The remaining studies [13, 24–29] used either the European Community criteria [41] or the American European Consensus Group criteria [40].

**Table 2:** Assessment of risk of bias

Risk of bias questions	Goodchild *et al.*, 2010 [13]	Gudbjörnsson *et al.*, 1993, study 1 [23]	Gudbjörnsson *et al.*, 1993, study 2 [23]	Hilditch *et al.*, 2008 [24]	Theander *et al.*, 2010 [25]	Tishler *et al.*, 1997 [26]	Usmani *et al.*, 2012 [27]	Van Oers *et al.*, 2010 [28]	Walker *et al.*, 2003 [29]
1. Was the sample representative of the target population?	N	N	N	N	Y	Y	Y	Y	N
2. Were study participants recruited in an appropriate way?	?	?	?	?	Y	Y	Y	?	Y
3. Was the sample size adequate?	N	Y	N	N	Y	Y	N	N	Y
4. Were the study subjects and the setting described in detail?	N	Y	N	N	Y	Y	Y	Y	Y
5. Was the data analysis conducted with sufficient coverage of the identified sample?	?	Y	N	Y	Y	Y	Y	Y	Y
6. Were objective, standard criteria used for the measurement of the condition?	Y	Y	Y	Y	Y	Y	Y	Y	Y
7. Was the condition measured reliably?	Y	Y	Y	Y	Y	Y	Y	Y	Y
8. Was there appropriate statistical analysis?	Y	Y	Y	Y	Y	Y	Y	Y	Y
9. Were all important confounding factors/ subgroups/differences identified and accounted for?	Y	?	?	?	Y	Y	?	?	Y
10. Were subpopulations identified using objective criteria?	N/A	N/A	N/A	N/A	N/A	N/A	N/A	N/A	N/A

Adapted with permission from Munn *et al.* [22]. The development of a critical appraisal tool for use in systematic reviews addressing questions of prevalence. Int J Health Policy Manag 2014;3:123–8.

N: no; N/A: not applicable; Y: yes; ?: unclear.

Several studies did not fully specify how their participants were recruited (including Gudbjörnsson *et al.* studies 1 and 2) [13, 23, 24, 28] and uncertainty remained for this item for these studies. The sample size was small (<40) for a number of studies (including Gudbjörnsson *et al.* study 2) [13, 23, 24, 27, 28] and these studies were scored as being at high risk of bias for this question.

Overall, three studies were deemed to be of high risk of bias (including Gudbjörnsson *et al.* study 2) [13, 23, 24], four at medium risk of bias (including Gudbjörnsson *et al.* study 1) [23, 27–29] and two at low risk of bias [25, 26].

## Prevalence of specific sleep difficulties in pSS

The main sleep outcomes are shown in Table 3. Perceived sleep disturbance (measured by sleep diary or patient-reported sleep questionnaires) was reported in four studies (including Gudbjörnsson *et al.* study 1) [13, 23, 26, 28]. Odds ratios (ORs) were calculated from the data provided by one study [26]. pSS patients scored significantly worse than healthy controls for this outcome. It was unclear whether overall there is a higher prevalence of sleep disturbance in pSS patients compared with other disease groups (OA, RA, SLE), as there were inconsistent findings between the studies.

**Table 3:** Differences in specific sleep outcomes between pSS patients and controls

	Study	Results	pSS
Perceived sleep disturbance	Goodchild *et al.*, 2010 [13]	pSS *vs* RA not significantly different for quality of sleep or feeling of refreshment	ND
	Gudbjörnsson *et al.*, 1993, study 1 [23]	44% pSS not feeling rested after sleep *vs* 9.8% RA (*P* < 0.001) and 15.3% HC (*P* < 0.01)	+
	Tishler *et al.*, 1997 [26]	Moderate/severe sleep disturbance 75% pSS. Significantly greater than OA and RA (*P* < 0.01)	+
	van Oers *et al.*, 2010 [28]	Significant differences in sleep disturbance (*P* < 0.001) between all groups (SLE, RA, HC, pSS). pSS highest median (6/15), HC lowest (2.3/15).	+
Time spent in bed	Gudbjörnsson *et al.*, 1993, study 2 [23]	pSS mean time in bed (500 min, range 444–532) similar to HC (range 419–514)	ND
	Theander *et al.*, 2010 [25]	pSS 45 min more in bed *vs* HC (8.24 *vs* 7.72 h; *P* = 0.048)	+
Total sleep time	Goodchild *et al.*, 2010 [13]	pSS mean 7 h asleep, similar to RA	ND
	Gudbjörnsson *et al.*, 1993, study 1 [23]	pSS mean of 5.2 h asleep (s.d. 1.90), significantly less than RA [6.8 h (s.d. 1.30), *P* < 0.05] and HC [7.2 h (s.d. 0.77), *P* < 0.0001]	+
	Gudbjörnsson *et al.*, 1993, study 2 [23]	pSS mean 358 min asleep (range 183–473), less than HC range (396–466 min)	+
	Hilditch *et al.*, 2008 [24]	No difference total sleep time pSS *vs* HC	ND
	Usmani *et al.*, 2012 [27]	No difference total sleep time pSS *vs* HC	ND
Sleep onset latency	Gudbjörnsson *et al.*, 1993, study 1 [23]	Mean time to fall asleep greatest in pSS [30 min (s.d. 52.49)] *vs* RA [21 min (s.d. 19.44)] and HC [19 min (s.d. 6.97)], difference not significant	ND
	Gudbjörnsson *et al.*, 1993, study 2 [23]	pSS mean 20 min to fall asleep (range 3–65), greater than HC range (1.5–13.6 min)	+
	Usmani *et al.*, 2012 [27]	pSS mean 22.8 min to fall asleep (range 14–40) *vs* 13.8 for HC (range 6–22) (*P* = 0.035)	+
Sleep efficiency	Goodchild *et al.*, 2010 [13]	pSS 84% sleep efficiency significantly less than RA 89.4% (*P* < 0.05)	+
	Gudbjörnsson *et al.*, 1993, study 2 [23]	pSS 70% mean sleep efficiency, well below control range (94–100%)	+
	Hilditch *et al.*, 2008 [24]	No difference in pSS group *vs* HC (combined mean 44% sleep efficiency)	ND
Number of night awakenings	Gudbjörnsson *et al.*, 1993, study 1 [23]	pSS woke mean 2.6 times, significantly more than RA (1.5) (*P* < 0.0001) and HC (1.0) (*P* < 0.05)	+
	Gudbjörnsson *et al.* 1993 Study 2 [23]	pSS woke mean 19 times (polysomnography) *vs* HC (range 1–7)	+
	Theander *et al.*, 2010 [25]	pSS awakenings [mean 2.7 (s.d. 0.17)] higher than HC [mean 1.7 (s.d. 0.18), *P* = 0.001]	+
Arousal index	Hilditch *et al.*, 2008 [24]	Trend of higher mean nocturnal arousals in pSS *vs* HC (*P* < 0.06)	ND
	Usmani *et al.*, 2012 [27]	No difference pSS *vs* HC (*P* = 0.18)	ND
Ventilatory measurements and sleep apnoea	Hilditch *et al.*, 2008 [24]	No difference pSS *vs* HC for upper airway collapsibility index and respiratory variables	ND
	Usmani *et al.*, 2012 [27]	Twice the frequency of apnoeas and hypoapnoeas in pSS *vs* HC (*P* = 0.032)	+
		Sleep apnoea 64% pSS *vs* 28% HC (*P* = 0.03)	
Daytime somnolence	Gudbjörnsson *et al.*, 1993, study 1 [23]	pSS significantly more daytime sleepiness *vs* HC (*P* < 0.001) or RA (*P* < 0.0001). pSS significantly more daytime naps [pSS 15.2% *vs* HC 0% (*P* < 0.01)], but not RA (21.4%)	+
	Theander *et al.*, 2010 [25]	pSS significantly worse daytime sleepiness [ESS; mean 9.5 (s.d. 5.2)] *vs* HC [mean 7 (s.d. 4.0)] (*P* = 0.003) and significantly more excessive sleepiness (15.3%) *vs* HC (11.9%) (*P* = 0.016)	+
	Usmani *et al.*, 2012 [27]	pSS significantly worse for daytime sleepiness [ESS; mean 10.1 (s.d. 5.82)] *vs* HC [mean 6.5 (s.d. 3.39), *P* = 0.014]	+
	Walker *et al.*, 2003 [29]	pSS daytime sleepiness significantly worse *vs* OA (OR 2.50, *P* = 0.01)	+

*P*-values are reported when provided in the published studies. +: favours controls; ESS: Epworth Sleepiness Scale; HC: healthy controls; ND: no difference.

Time spent in bed was assessed in two studies with conflicting findings. One study objectively assessed this outcome with polysomnography (Gudbjörnsson *et al.* study 2 [23]) and the other measured time in bed subjectively with a patient-reported sleep questionnaire [25]. However, the study that reported no difference for this outcome between pSS patients and healthy controls (Gudbjörnsson *et al.* study 2) [23] had very small numbers and took the measurements in a laboratory. The polysomnography study protocol and environment may have influenced how long a participant remained in bed. Therefore it is unclear whether pSS patients spend a longer time in bed compared with other populations.

We identified five separate studies that examined total sleep duration, including the Gudbjörnsson *et al.* studies 1 and 2 [13, 23, 24, 27]. Three small studies [13, 24, 27] compared a total of 53 pSS patients with RA patients (*n* = 25) [13] or healthy controls (*n* = 26) [24, 27]. They found no significant differences between the groups in terms of total sleep time. However, Gudbjörnsson *et al.* study 1 [23] compared 40 people with pSS with 42 people with RA and 60 healthy controls. They found that people with pSS reported significantly less sleep than the comparators as measured by sleep diaries (40 min–1 h 45 min less), while in their smaller polysomnography study (study 2) they found that pSS patients experienced 1 h 18 min–2 h less sleep than healthy controls.

Three studies examined the proportions of time spent in each of the stages of sleep between pSS patients and controls (including Gudbjörnsson *et al.* study 2) [23, 24, 27]. Two found that pSS patients spent more time in stage 1 sleep than controls (Gudbjörnsson *et al.* study 2) [23, 24]. However, Usmani *et al.* [27] found no such difference. None of the studies found between-group differences for other stages of sleep.

Sleep onset latency (time taken to fall asleep) was not significantly different between pSS patients, RA patients and healthy controls using self-reported methods in one study (Gudbjörnsson *et al.* study 1) [23], although the authors did not make a direct comparison between the pSS and control groups. However, two studies (including Gudbjörnsson *et al.* study 2) [23, 27] involving objective testing of this outcome (polysomnography) did find sleep onset latency to be greater in pSS patients (mean 20–22 min) compared with controls (mean 13.8 [27], range 1.5–13.6 min [23]).

Sleep efficiency (percentage of time spent in bed asleep) was identified as reduced in people with pSS in two studies (including Gudbjörnsson *et al.* study 2) [13, 23], both of which used objective measures. In a third study [24] with very low numbers, the sleep efficiency was very poor for both groups. However, this could be due to the nasal mask that participants wore and the regular negative pressure pulses used to measure airway collapsibility, which may have interfered with their sleep. Thus the environment was not ideal to examine sleep efficiency in this study.

All studies that examined the number of night awakenings found that these were increased in pSS patients (Gudbjörnsson *et al.* study 2 and Theander *et al.*) [23, 25] (see Table 3). That being said, the polysomnography studies that report an arousal index (number of times sleep is interrupted) found no difference between pSS patients and comparison groups [24, 27].

## Factors associated with disturbed sleep

A number of studies examined specific reasons for waking in the night. Theander *et al.* [25] noted that 13% of their pSS group reported sicca symptoms that disturbed their sleep, compared with none of their controls.

Hilditch *et al.* [24] found that nocturnal oral dryness did not differ significantly between pSS patients and controls, which is surprising, but due to their very low numbers, could be a type II error. The same authors found that saliva surface tension showed no difference between the groups in the early morning but was significantly higher in the pSS group in the late evening.

Nocturnal pain and disturbed sleep was more common in pSS compared with controls and RA patients (Gudbjörnsson *et al.* study 1 and Theander *et al.*) [23, 25]. Gudbjörnsson *et al.*study 1 [23] reported that 54% of their pSS group experienced nocturnal pain compared with 37% of their RA group (*P* < 0.01) and 0% of their healthy control group (*P* < 0.0001). Theander *et al.* [25] found that nocturnal pain that disturbed sleep was present in 19% of their pSS group, which was >9% of those in the control group, although this difference was not significant (*P* = 0.07).

There was conflicting evidence from two studies for nocturia disturbing sleep in pSS patients. Walker *et al.* [29] investigated nocturia and found no difference between pSS patients and an OA population for the occurrence of this symptom (OR 0.93, *P* = 0.85). Conversely, Theander *et al.* [25] found that 53% of their pSS participants experienced nocturia that disturbed sleep compared with 26% of their healthy controls (*P* = 0.001).

### Autonomic symptoms

Nocturnal autonomic symptoms were only investigated by Gudbjörnsson *et al.* study 1 [23], which found 20% of the pSS participants in this study reported experiencing nocturnal sweating, which was greater than their RA comparison group (12%, NS) and their healthy controls (2%, *P* < 0.01). Palpitations at night were reported in 5% of their pSS group, which were not present in either their RA or healthy control groups.

## Presence of co-morbid sleep disorders

In Theander *et al.*’s study [25], 2 of their 72 patient pSS cohort self-reported a diagnosis of narcolepsy, compared with none of their controls, but this was not reported as an outcome in any of the other included studies.

Using polysomnography, one study noted the occurrence of obstructive apnoeas and hypopnoeas were double in their pSS group compared with healthy controls [27]. In this study, continuous positive airway pressure (CPAP) treatment was offered to 8 of 28 pSS study participants who were identified as having severe sleep apnoea (with an apnoea-hypnoea index score ⩾40). Five participants accepted the treatment and significant improvements were demonstrated both in their Epworth Sleepiness Scale (ESS) scores and fatigue scores at 2–6 months after commencing CPAP treatment. 

However, another study [24] investigated upper airway collapsibility and found no difference in both the upper airway collapsibility index and a range of respiratory variables between their pSS and control groups, but this could be due to the study being underpowered.

## Daytime somnolence

Four studies identified increased daytime sleepiness in patients with pSS compared with healthy controls. Gudbjörnsson *et al.* study 1 [23] found their pSS patients were sleepy in the daytime five times more frequently than RA controls and almost three times more frequently than healthy controls. Theander *et al.* [25], Usmani *et al.* [27] and Walker *et al.* [29] reported that ESS scores were significantly higher in pSS patients than in controls.

## Discussion

### Findings of the review

We have found that subjective and objective sleep disturbances are more common in pSS patients. Further research is needed to examine the differences between pSS patients and other disease groups.

There were inconclusive findings regarding whether pSS patients spend more time in bed than comparative groups, however, if they do spend longer in bed, it is likely that this is due to the sleep disturbances and night awakenings they experience. Because of the conflicting findings in this review, further studies are needed to confirm whether pSS patients have a short sleep duration compared with other groups. However, pSS patients do seem to experience more frequent nocturnal awakenings than other groups. Despite this finding, the arousal index scores were not found to be greater for pSS patients in the studies that examined this outcome. One reason could be due to low numbers of participants in these studies. However, an alternative suggestion is that the pSS patients awaken more frequently during these arousals due to their symptoms, such as dryness, pain and autonomic symptoms. A further possibility is that pSS patients may demonstrate high-frequency electroencephalographic activity throughout the night that may influence their perception of sleep and wakefulness. Further investigations are required to test this.

Sicca symptoms did disturb sleep in one study [25] and potential interventions to reduce these symptoms and thus improve sleep will be discussed shortly. Pain is another symptom that is more common in pSS patients during the night. Segal *et al.* [4] observed that sleep quality is reduced as pain increases. Thus if pain is reduced, sleep quality may improve.

There were conflicting findings regarding the symptom of nocturia in pSS patients. Since pSS patients regularly drink to ease the symptoms of their dryness, needing the toilet during the night could be a natural consequence of this.

Although autonomic symptoms were only reported in one included study, there is a greater prevalence of these symptoms in pSS patients [3] and it is logical that these symptoms, which can include palpitations, dizziness and sweats, may interfere with sleep.

There does seem to be an increased prevalence of obstructive sleep apnoea in pSS patients, although further studies are needed to reproduce this finding. The ESS can be used as a screening tool to identify patients who are at risk of obstructive sleep apnoea [42] and these patients should be referred for further investigations.

This review has demonstrated that daytime sleepiness is a problem in pSS patients. Daytime sleepiness correlates with reduced quality of life [10], fatigue [25, 29], autonomic dysfunction [3] and functional impairment [6]. Furthermore, patients who are functionally impaired have significantly greater ESS scores than those who experience no functional disability [6].

### Potential interventions for sleep disturbances in pSS

Interventions that address the perception of poor sleep without the necessity for objective verification include addressing unhelpful beliefs surrounding sleep, addressing sleep efficiency and prescribing time in bed. These are all components of a Cognitive Behavioural Therapy for Insomnia (CBT-I) intervention [43]. CBT-I is considered a first-line treatment for insomnia associated with a medical condition [44] and is an effective intervention in other long-term conditions [45], therefore it could be beneficial in pSS. Time in bed and sleep efficiency are both addressed in the sleep restriction component of a CBT-I intervention and this may be a useful way of addressing longer time spent in bed awake in this patient group and lead to improved sleep. Further studies of CBT-I and various modes of delivering this intervention are therefore warranted in this patient group.

Nocturnal humidification and artificial saliva sprays may ease nocturnal sicca symptoms and decrease sleep disturbances in pSS patients and are unlikely to contribute to bladder disturbances during the night. Although a humidification device did seem to be a promising intervention in an excluded study [30], further appropriately powered studies comparing nocturnal humidification devices in pSS with controls are required to demonstrate efficacy.

For autonomic symptoms that interfere with sleep, appropriate interventions addressing these symptoms, such as water bolus treatment during the day [46], may also help to improve sleep, particularly if these symptoms are regularly experienced during the night. Further research is required to demonstrate the efficacy of interventions for dysautonomia in pSS, such as blood pressure dysregulation, on sleep outcomes.

### Further considerations

A more detailed sleep assessment, including polysomnography, may be beneficial for this group when considering the level of sleep apnoea reported in this population. Moreover, polysomnography will afford a closer examination of other objectively verifiable sleep disorders that may influence sleep (e.g. narcolepsy, periodic limb movement disorder, restless legs syndrome and hypersomnolence disorders). If severe sleep apnoea is identified in pSS patients, CPAP treatment should be offered [47].

Pain is another symptom that can interrupt sleep. pSS patients who experience pain that is interfering with their sleep should be offered appropriate pain management interventions [4]. CBT-I is efficacious in improving sleep duration, continuity and perceived quality in chronic pain patients with co-morbid insomnia and CBT-I with an additional pain component is feasible [45]. A pain adjunct to a CBT-I intervention may therefore improve sleep in pSS patients with chronic pain. Interventions targeting sleep disturbances in pSS may improve daytime sleepiness and fatigue, which could result in increased functional capacity and quality of life.

There are some limitations to this review. First, although we did not specifically investigate potential causes of sleep disturbances, we uncovered several potential contributing factors from within the included studies. However, there may be further potential complications in pSS that might play a role in sleep disturbance, such as gastro-oesophageal reflux [48]. Further work needs to be done to determine the causes of sleep disturbances in this patient group. Second, although we did not set out to investigate specific interventions for sleep disturbances, we identified some uncontrolled studies of interventions for sleep in pSS. There may therefore be further studies of interventions for sleep disturbances in pSS uncovered by our search. However, a recent systematic review of all non-pharmacological interventions for pSS did not identify any randomized controlled trials for sleep difficulties in this patient group [49]. Furthermore, recent meta-analysis of 23 studies determined that CBT-I was efficacious in reducing sleep disturbances and improving sleep quality in patients with insomnia secondary to a co-morbid condition [50].

This review included a total of 350 pSS patients in nine separate studies. Only two studies (with 142 pSS patients) were deemed to be at low risk of bias. This highlights the paucity of high-quality research into sleep disturbances in pSS patients.

## Conclusion

From the included studies in this review, we found an increased prevalence of sleep disturbances in pSS patients compared with controls, including daytime somnolence, subjective sleep disturbances (including disturbance due to dryness symptoms) and increased occurrence of night awakenings. Sleep apnoea may be more common, but further polysomnography studies are required to confirm this.

Although we did not set out to investigate interventions, logic dictates that CBT-I for sleep disturbances and night awakenings and nocturnal humidifiers for nocturnal sicca symptoms would be beneficial in this patient group. However, further studies are required to confirm their effectiveness in pSS. Due to the variable quality of the included studies, the mix of outcomes assessed within these studies and the overall low numbers of patients included within them, we recommend further studies to add to the body of pSS sleep prevalence literature. Finally, in the presence of sleep difficulties in pSS patients, primary sleep disorders should be screened for and treated appropriately.

*Funding*: This work was funded by Arthritis Research UK (Grant 20169) and a Research Career Development Grant from the United Kingdom Occupational Therapy Research Foundation.

*Disclosure statement*: The authors have declared no conflicts of interest.

## Supplementary data

Supplementary data are available at *Rheumatology* Online.

## Supplementary Material

Supplementary Data
