# Supplementary material for: An investigation into the prevalence of sleep disturbances in primary Sjögren’s syndrome: a systematic review of the literature
Source: Rheumatology (Oxford). 2016 Dec 24;56(4):570–80. doi: 10.1093/rheumatology/kew443 (PMC5410987; doi:10.1093/rheumatology/kew443)
Supplement: Supplementary Data [file kew443_Supp.docx]

### SUPPLEMENTARY DATA

### Supplementary Table S1: Search strategy

| The following databases were searched from inception to September 2015: PubMed; Medline (OVID); Embase (OVID); PsychINFO (OVID) and Web of Science using the following search string: |
| --- |
| 1. sjogrens syndrome/ |
| 1. sjogrens syndrome.mp. |
| 1. 1 or 2 |
| 1. exp sleep/ |
| 1. 3 and 4 |

###### Supplementary Table S2: Assessment of risk of bias questions demonstrating the criteria used by the reviewers to reach a decision

| **Risk of Bias Questions included in the Joanna Briggs Institute Prevalence Critical Appraisal Tool** | **Clarification notes agreed by the reviewers (KH and KD) and used to reach a decision for the purpose of this review** |
| --- | --- |
| 1. Was the sample representative of the target population? | Accepted and validated diagnostic criteria for PSS and good range of patients – i.e. all female cohorts are scored no |
| 2. Were study participants recruited in an appropriate way? | Studies recruiting consecutive clinic patients or using a random sampling frame were scored yes. Where recruitment strategy was not made explicit in the text – the study was scored unclear for this question. |
| 3. Was the sample size adequate? | Suitable sample size ≥40 required for study to be scored yes |
| 4. Were the study subjects and the setting described in detail? | Studies were scored yes if they provided a table of baseline characteristics for all groups |
| 5. Was the data analysis conducted with sufficient coverage of the identified sample? | Up to a 10% drop out of participants over one year was permitted, ≥11% drop out over one year were scored no |
| 6. Were objective, standard criteria used for the measurement of the condition? | Use of validated questionnaires/methods of sleep assessment were required to score yes |
| 7. Was the condition measured reliably? | Studies were scored yes if the measures were used appropriately |
| 8. Was there appropriate statistical analysis |  |
| 9. Were all important confounding factors/ subgroups/differences identified and accounted for? |  |
| 10. Were subpopulations identified using objective criteria? |  |

Adapted with permission from [1] Munn Z et al. The development of a critical appraisal tool for use in systematic reviews addressing questions of prevalence. International Journal of Health Policy and Management. 2014;3(3):123-8.

**Supplementary table 3: Table showing studies excluded from the review with reasons for exclusion**

| Study | Reason for exclusion |
| --- | --- |
| Abad (2008) [2] | Review paper |
| Aiyappan (2013) [3] | Conference abstract |
| Gauba (2014) [4] | PSS data not reported separately for PSS patients |
| George and Pope (2011) [5]  Hackett (2012) [6]  Hay and Morton (2006) [7] | The study investigated minimally important differences in patient reported outcomes  Did not compare sleep outcomes with controls  Did not compare sleep outcomes with a non PSS control group |
| Hening (2008) [8] | Review paper |
| Heran (2010) [9] | Conference abstract |
| Hsiao (2015) [10]  Kaarela (1992) [11] | The study population had a sleep disorder which was being investigated for an increased risk of PSS (not vice versa)  Did not compare sleep outcomes with a non PSS control group |
| Leeuwen (2013) [12]  Lendrem (2014) [13] | Conference abstract  Did not compare sleep outcomes with controls |
| Newton (2011) [14] | Conference abstract |
| Segal (2013) [15] | Did not compare sleep outcomes with a non PSS control group |
| Streitzel (2011) [16] | PSS data not reported separately for PSS patients |
| Thie (2002) [17] | Review paper |
| Tufik (2013) [18] | Letter |
| Usmani (2013) [19] | Letter |
| Vissink (1986) [20] | Review paper |

## References

1 Munn Z, Moola S, Riitano D, Lisy K. The development of a critical appraisal tool for use in systematic reviews addressing questions of prevalence. International journal of health policy and management. 2014;3(3):123-8.

2 Abad VC, Sarinas PS, Guilleminault C. Sleep and rheumatologic disorders. Sleep Med Rev 2008;12(3):211-28.

3 Aiyappan V, Catcheside P, McEvoy D, et al. A randomised placebo controlled cross-over trial of airway humidification in patients with primary sjogrens syndrome (PSS) and its effect on sleep apnea and sleep quality. Sleep and Biological Rhythms 2013;11:48.

4 Gauba V, Curtis ZJ. Sleep position and the ocular surface in a high airflow environment. Saudi journal of ophthalmology : official journal of the Saudi Ophthalmological Society 2014;28(1):66-8.

5 George A, Pope JE. The minimally important difference (MID) for patient-reported outcomes including pain, fatigue, sleep and the Health Assessment Questionnaire Disability Index (HAQ-DI) in primary Sjogren's syndrome. Clinical and Experimental Rheumatology 2011;29(2):248-53.

6 Hackett KL, Newton JL, Frith J, et al. Impaired functional status in primary Sjogren's syndrome. Arthritis Care and Research 2012;64(11):1760-4.

7 Hay KD, Morton RP. Optimal nocturnal humidification for xerostomia. Head and Neck-Journal for the Sciences and Specialties of the Head and Neck 2006;28(9):792-6.

8 Hening WA, Caivano CK. Restless legs syndrome: A common disorder in patients with rheumatologic conditions. Seminars in Arthritis and Rheumatism 2008;38(1):55-62.

9 Heran S, Hlavac M, Hilditch C, et al. Increased sleep disordered breathing in Sjogren's syndrome. Sleep and Biological Rhythms 2010;8:A29.

10 Hsiao Y-H, Chen Y-T, Tseng C-M, et al. Sleep Disorders and Increased Risk of Autoimmune Diseases in Individuals without Sleep Apnea. Sleep 2015;38(4):581-6.

11 Kaarela K, Mutru O. Xerostomia in Sjogren's syndrome treated with Sali-Synt. A double-blind cross-over trial. Scandinavian journal of rheumatology 1982;11(1):39-40.

12 Leeuwen NV, Bossema ER, Kruize AA, Bootsma H, Bijlsma JW, Geenen R. Sleep and nightly oral dryness as potential therapeutic targets to reduce fatigue in sjogren's syndrome. Psychosomatic Medicine 2013;75 (3):A156-A7.

13 Lendrem D, Mitchell S, McMeekin P, et al. Health-related utility values of patients with primary Sjogren's syndrome and its predictors. Ann Rheum Dis 2014;73(7):1362-8.

14 Newton J, Powell D, Mitchell S, Griffiths B, Bowman S, Ng WF. Potentially treatable symptoms in primary sjogren's syndrome-associated fatigue. Rheumatology 2011;50:iii104-iii5.

15 Segal BM, Pogatchnik B, Henn L, Rudser K, Sivils KM. Pain Severity and Neuropathic Pain Symptoms in Primary Sjogren's Syndrome: A Comparison Study of Seropositive and Seronegative Sjogren's Syndrome Patients. Arthritis Care & Research 2013;65(8):1291-8.

16 Strietzel FP, Lafaurie GI, Bautista Mendoza GR, et al. Efficacy and Safety of an Intraoral Electrostimulation Device for Xerostomia Relief A Multicenter, Randomized Trial. Arthritis and Rheumatism 2011;63(1):180-90.

17 Thie NM, Kato T, Bader G, Montplaisir JY, Lavigne GJ. The significance of saliva during sleep and the relevance of oromotor movements. Sleep Med Rev 2002;6(3):213-27.

18 Tufik SB, Bennedsen L, Andersen ML, Tufik S. The interaction of Sjogren's syndrome, gastroesophagel reflux and sleep. Sleep Medicine 2013;14(2):222.

19 Usmani ZA, Hlavac M, Rischmueller M, et al. Sleep disordered breathing in patients with primary Sjogren's syndrome: a group controlled study. Sleep Medicine 2012;13(8):1066-70.

20 Vissink A, Panders AK, Gravenmade EJ, Vermey A. Treatment of oral symptoms in Sjogren's syndrome. Scandinavian journal of rheumatology. Supplement 1986;61:270-3.
